# A Comprehensive View on the Quercetin Impact on Colorectal Cancer

**DOI:** 10.3390/molecules27061873

**Published:** 2022-03-14

**Authors:** Andreea-Adriana Neamtu, Teodor-Andrei Maghiar, Amina Alaya, Neli-Kinga Olah, Violeta Turcus, Diana Pelea, Bogdan Dan Totolici, Carmen Neamtu, Adrian Marius Maghiar, Endre Mathe

**Affiliations:** 1 Doctoral School of Biomedical Sciences, University of Oradea, 410087 Oradea, Romania; aneamtu94@gmail.com (A.-A.N.); teodormaghiar@yahoo.com (T.-A.M.); 2 Faculty of Medicine, “Vasile Goldis” Western University of Arad, 310045 Arad, Romania; violeta.turcus@uvvg.ro (V.T.); totolici.bogdan@uvvg.ro (B.D.T.); endre.mathe@agr.unideb.hu (E.M.); 3 Department of Surgical Disciplines, Faculty of Medicine and Pharmacy, University of Oradea, 410081 Oradea, Romania; 4 Doctoral School of Animal Science, Faculty of Agricultural and Food Sciences and Environmental Management, University of Debrecen, H-4032 Debrecen, Hungary; aleya.amina@gmail.com; 5 SC PlantExtrAKT SRL, 407059 Rădaia, Romania; neli.olah@plantextrakt.ro; 6 Faculty of Pharmacy, “Vasile Goldis” Western University of Arad, 310045 Arad, Romania; 7 National Institute for Economic Research “Costin C. Kiritescu” of the Romanian Academy/Centre for Mountain Economy (CE-MONT), 725700 Suceava, Romania; 8 Faculty of Medicine and Pharmacy, University of Oradea, 410081 Oradea, Romania; diana_pelea@yahoo.com; 9 Institute of Nutrition, Faculty of Agricultural and Food Sciences and Environmental Management, University of Debrecen, H-4032 Debrecen, Hungary

**Keywords:** quercetin, flavonol, polyphenol, colorectal cancer, PI3K/AKT, Wnt/β-catenin, MAPK, p53, NF-κB

## Abstract

Colorectal cancer (CRC) represents the third type of cancer in incidence and second in mortality worldwide, with the newly diagnosed case number on the rise. Among the diagnosed patients, approximately 70% have no hereditary germ-line mutations or family history of pathology, thus being termed sporadic CRC. Diet and environmental factors are to date considered solely responsible for the development of sporadic CRC; therefore; attention should be directed towards the discovery of preventative actions to combat the CRC initiation, promotion, and progression. Quercetin is a polyphenolic flavonoid plant secondary metabolite with a well-characterized antioxidant activity. It has been extensively reported as an anti-carcinogenic agent in the scientific literature, and the modulated targets of quercetin have been also characterized in the context of CRC, mainly in original research publications. In this fairly comprehensive review, we summarize the molecular targets of quercetin reported to date in in vivo and in vitro CRC models, while also giving background information about the signal transduction pathways that it up- and downregulates. Among the most relevant modulated pathways, the Wnt/β-catenin, PI3K/AKT, MAPK/Erk, JNK, or p38, p53, and NF-κB have been described. With this work, we hope to encourage further quests in the elucidation of quercetin anti-carcinogenic activity as single agent, as dietary component, or as pharmaconutrient delivered in the form of plant extracts.

## 1. Epidemiology and Aetiology of Colorectal Cancer

Colorectal cancer (CRC), responsible for 10% of diagnosed cancers and 9.4% of cancer deaths worldwide in 2020, is the third type of cancer in incidence and second in mortality by anatomical location in both sexes [1]. In absolute numbers, this translates to 1.9 million newly diagnosed cases in 2020, and a prediction of 2.5 million cases for 2035 [1,2].

The influence of westernized dietary habits and lifestyle on CRC development is furthermore supported by statistics correlating CRC occurrence with the Human Development Index (HDI), with approximately fourfold higher incidence and twofold higher mortality of CRC in transitioned countries than in transitioning ones [1]. Moreover, this correlation is reinforced by the aetiology of CRC, claiming approximately 70% of the cases to be of sporadic origin, linked to diet and environmental influences, while only 5–10% are accounted for as hereditary germ-line mutations in genomic studies [3,4,5,6,7,8]. Concerning the remaining CRC diagnosed patients, family history has been claimed; however, there is to date no molecular evidence supporting hereditary transmission of the responsible mutated genes [3,4,5,6,7,8].

## 2. Phytochemicals—Potential Benefits

In correlation with the epidemiology and etiology of CRC, studies have established that diet is a risk determinant in the development of the disease, linking the consumption of fruits, vegetables, whole grains, and nuts to a decrease the development of sporadic CRC through protective mechanisms [9,10,11,12,13,14].

Whilst in a broad sense, phytochemicals, also referred to as phytonutrients, represent chemicals produced by plants, this rudimentary definition has nowadays been shifted towards non-nutritive secondary metabolites of plants, often acting in the human body as modulators of critical cellular signaling pathways, inducing health improvement [6,15]. Through clinical and pre-clinical research, the anti-carcinogenic activity of phytochemicals has been postulated through inhibition of mitosis, induction of apoptosis and excretion of carcinogens [16], in addition to their more established functions as antioxidants and anti-inflammatory agents [17]. Thus, it is plausible that administration of phytochemicals, in tandem with cancer treatments, could improve the prognosis of the disease and constitute a new approach in the management of patients with CRC pathology [18].

Nevertheless, with more than 10,000 class representatives identified to date, and many more remaining to be discovered, individual compounds among phytochemicals vary in chemical structures, their mechanisms of action, and their metabolites [19]. Therefore, this subject constitutes a broad avenue to be explored, as a single compound action, alongside the synergistic effects of a multitude of compounds [20].

## 3. Quercetin

**Figure 1:**
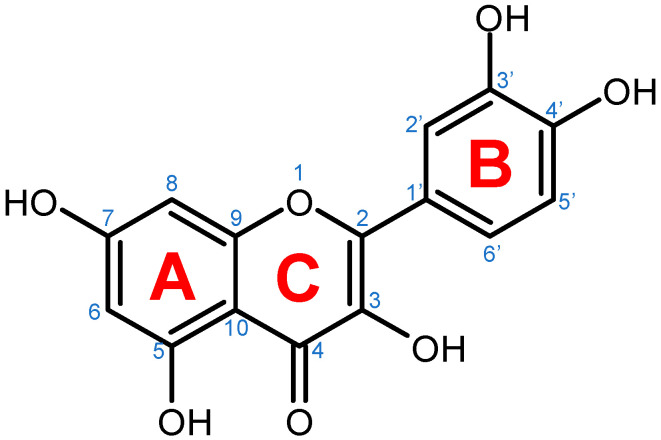
Chemical structure of quercetin with numbered carbon atoms (blue) and marked rings (red) on the general flavonoid backbone structure.

Quercetin is a plant pigment and secondary metabolite, a polyphenolic flavonoid phytochemical with a well-characterized antioxidant activity [21,22]. Chemically, it is a pentahydroxyflavone, using as a backbone the flavone structure C6(A-ring)-C3(C-ring)-C6(B-ring) (Figure 1) [22]. The official IUPAC name of the compound is 2-(3,4-dihydroxyphenyl)-3,5,7-trihydroxy-4H-chromen-4-one and the chemical structure C_15_H_10_O_7_ [23,24]. The molecular weight of quercetin is 302,2 g/mol, and in its purified form it is a yellow-colored crystalline solid at room temperature, with poor water solubility, but increased solubility in alkaline aqueous solutions and alcohols, having a low acute toxicity level through oral exposure at LD_50_ 161 mg/kg [22,25]. The average daily intake is approximately 25 mg according to the US Department of Health and Human Services and studies carried out in Japan, France, and Finland [26,27,28,29,30], due to consumption of major food sources such as onions, asparagus, and berries, while reduced quantities are acquired from various other plants (Table 1) [31,32].

### 3.1. Quercetin Biosynthesis in Plants

In plants, as an important secondary metabolite, quercetin facilitates vital physiological processes, such as seed germination, photosynthesis, growth and development, and pollen production, alongside its powerful antioxidant activity that provides tolerance against both biotic and abiotic stressors [22]. The synthesis quercetin is organ-restricted and tightly regulated due to particular needs for specific flavonols under certain stress conditions [22,33,34].

**Figure 2:**
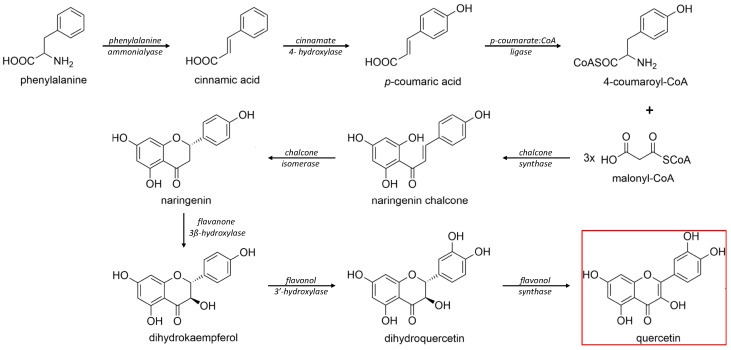
Schematic biosynthesis mechanism of quercetin from phenylalanine. Catalytic enzymes responsible are denoted in italics over the arrows. Quercetin, the final product, is marked with a red rectangular border.

The biosynthesis of quercetin (Figure 2) uses the aromatic α-essential amino acid phenylalanine, synthesized through the shikimic acid pathway, as starting molecule and follows the phenyl propanoid metabolic pathway [22]. Under the activity of the enzyme phenylalanine ammonialyase, a linking enzyme between the primary and secondary plant metabolism, L-phenylalanine is converted into trans-cinnamic acid. The latter is thereafter turned into p-coumaric acid in a reaction catalyzed by the enzyme cinnamate 4-hydroxylase, a cytochrome P450 monooxygenase in plants [22,33,35,36,37,38]. Through the functional moiety carboxylic acid, the p-coumaric acid is ligated by the p-coumarate:CoA ligase to CoA, forming the intermediary product 4-coumaroyl-CoA. Furthermore, 4-coumaroyl-CoA is linked to three molecules of malonyl-CoA, derived from fatty acid metabolism, with the help of the chalcone synthase enzyme. This reaction yields the essential A- and B-rings of the flavonoid skeleton (Figure 1) as naringenin chalcone. For the construction of the heterocyclic C-ring, chalcone isomerase acts on naringenin chalcone yielding the flavanone naringenin [6,22,33,36,37]. Naringenin is further hydroxylated through flavanone 3β-hydroxylase leading to the synthesis of dihydrokaempferol. Through a second consecutive hydroxylation reaction, dihydrokaempferol turns into dihydroquercetin with the help of the catalyst flavonol 3′-hydroxylase. Lastly, flavonol synthase acts on dihydroquercetin, yielding the final product in this synthesis, namely, the flavonol quercetin [22,33,36,37].

Structural variations in quercetin derivatives can be attributed to the exchange of the hydrogen ions with other groups, including hydroxyl, methoxyl, and glycosyl (Table 2) [39]. For example, among the most important derivatives of quercetin, isoquercetin (quercetin 3-O-β-D-glucoside) contains a glucose moiety attached to the 3-OH group on the C-ring of quercetin. In terms of biological activity, quercetin and its derivatives possess distinct efficiencies and activities due to the presence modifications at significant positions in the quercetin molecule [40].

### 3.2. Regulation of Quercetin Biosynthesis in Plants

The synthesis of quercetin is heavily linked to the general flavonoid biosynthetic pathway in plants. Therefore, it is dependent on the transcription regulation of all pathways leading up to anthocyanins, as they represent products that in their biosynthesis require the most steps of the flavonoid metabolism [42]. Transcription factors such as the basic helix–loop–helix (bHLH), R2R3-MYB, and the WD40 proteins lead to the activation, and differential temporal and spatial expression of the structural genes necessary in the biosynthesis [22,42,43]. Furthermore, concomitant increase in the synthesis of quercetin also occurs alongside the synthesis of lignin, mainly due to expression upregulation of the same set of enzymes pertaining to the phenylpropane metabolic pathway, pathway responsible for the synthesis of both molecules [44]. Moreover, several studies identify influences of environmental stressors, such as light and soil composition, as regulators of flavonoid synthesis [22,42]. The UV-B radiation and, consequently, the photosynthetic photon flux impact the biosynthesis of quercetin, with stronger photon fluxes being positively correlated with higher amounts of synthesized quercetin [45,46]. The stress induced by a high salinity soil has also been correlated with increased quercetin biosynthesis, consistent with an evolutive stress response reaction, resulting in a decrease in total flavonoid and an increase in flavonol content (quercetin and kaempferol) [47,48].

### 3.3. Quercetin Metabolism in Humans 

A substantial interindividual variability in quercetin bioavailability was reported in literature, attributed to various factors ranging from health status, body mass index, dietary adaptation, gut microbiota composition, to genetic polymorphisms [49].

**Figure 3:**
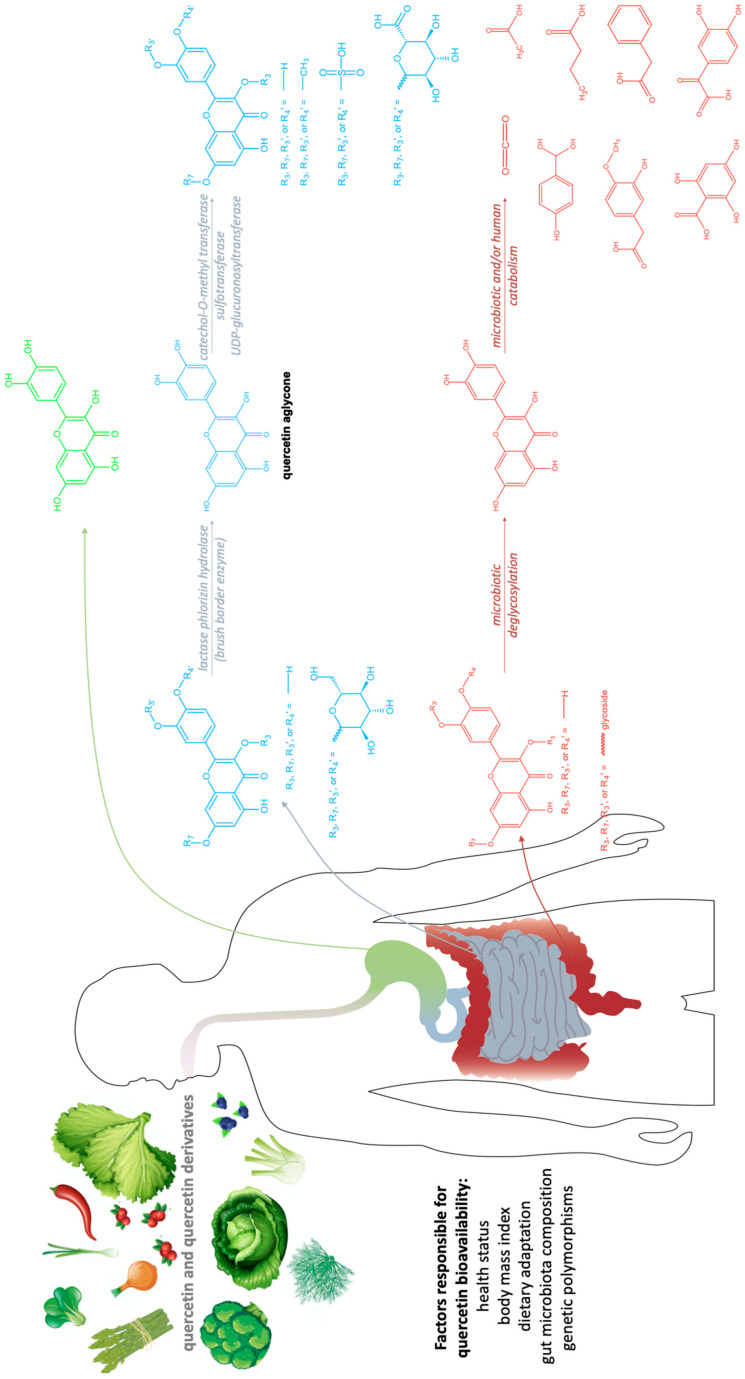
Quercetin metabolism in the gastro-intestinal tract. Green—stomach absorption; blue—small intestine absorption; red—large bowel absorption.

While the primary site of quercetin absorption is the small intestine [50,51,52], there is a small fraction observed to be already absorbed in the stomach, and a fraction that reaches the colon (Figure 3) [53]. Dietary sources provide a mixture of quercetin and quercetin derivatives (Table 2). Unfortunately, humans only absorb the aglycone, the un-modified quercetin [54], which is less accessible in the gastrointestinal tract than its substituted derivatives due to lower aqueous solubility, however, it starts to be absorbed in the stomach [55,56,57]. In order to be absorbed, the sugar moieties linked to the flavonol need to be removed by enzymes such as the lactase phlorizin hydrolase, a brush border enzyme specific for glucose and found in the small intestine [54]. Furthermore, in the case of quercetin derivatives containing any other glycosides, the aid of microbiota is necessary in the deglycosylation process [58,59,60,61].

Post enterocytic absorption, quercetin undergoes transformations such as glucuronidation (enzyme: UDP-glucuronosyltransferase; acts on more than 75% of the quercetin that reaches the bloodstream), methylation (enzyme: catechol-O-methyl transferase; acts on about 20% of the quercetin that reaches the bloodstream as tamarixetin and isorhamnetin), or sulfation (enzyme: sulfotransferase), in order to increase its aqueous solubility and be transferred to the bloodstream or to be excreted back to the intestinal lumen [60,62,63,64]. At inflammation sites, quercetin glucuronides could be deconjugated for a more potent anti-inflammatory, antioxidant, and analgesic response [65,66,67,68]. Rutin is an example of quercetin derivative which needs the aid of gut microbiota to be deglycosylated in the colon, through the enzymatic activity of α-rhamnosidases and β-glucosidases [69,70,71,72,73]. Then, colonocytes absorb the aglycone, turning it into lower-molecular-weight phenolic species through catabolic reactions, or follow the modifications pattern of enterocytes, releasing the product in the bloodstream [49]. Among the small molecules that can be obtained from quercetin, it is worth mentioning 2(3,4-dihydroxyphenyl)-2-oxoacetic acid, 3-methoxy-4-hydroxy-phenylacetic acid, p-hydroxybenzoic acid, phenylacetic acid, 2,4,6-trihydroxybenzoic acid, butyrate, acetate, and finally CO_2_, which can also be degraded through the catabolism of the microbiota [49]_._

## 4. Quercetin and Its Derivatives—Mechanisms of Action in CRC

**Figure 4:**
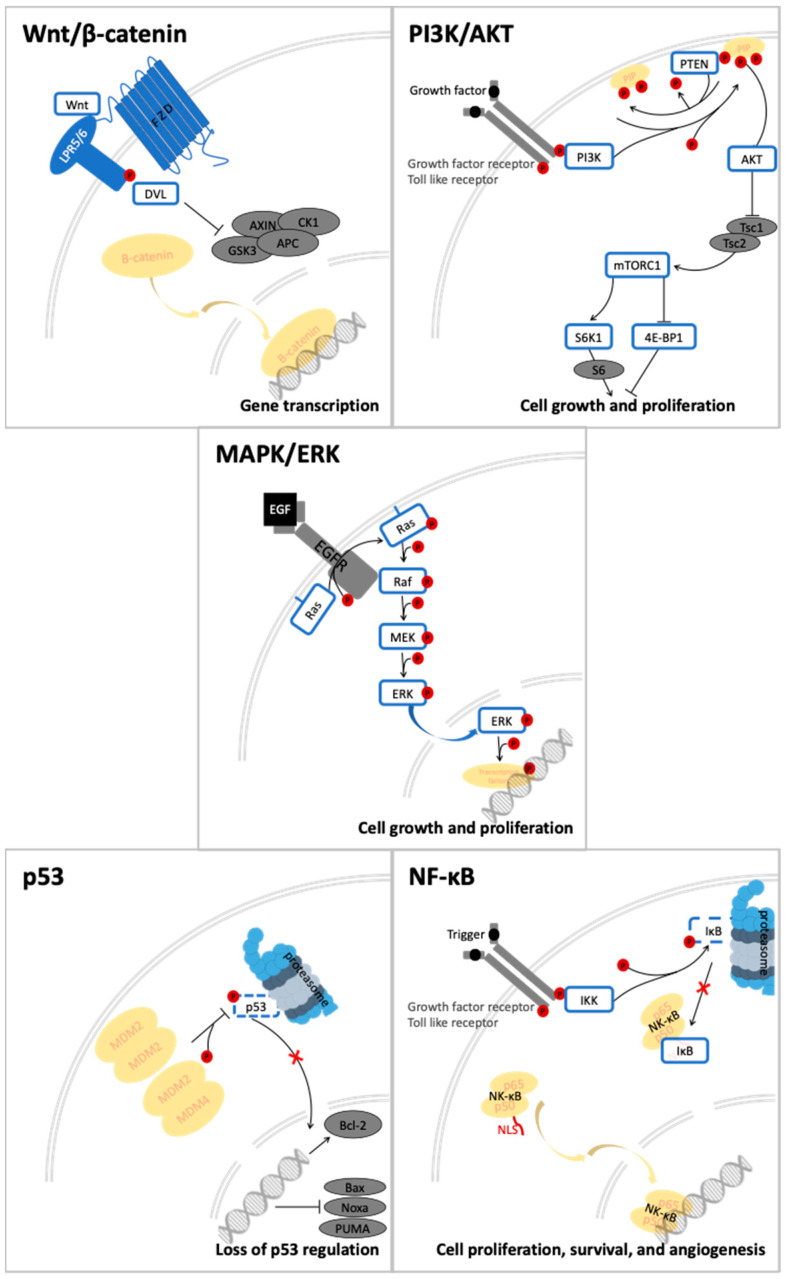
Signal transduction pathways in CRC that are modulated by quercetin: Wnt/β-catenin, PI3K/AKT, MAPK (using MAPK/ERK as an example for the phosphorylation cascade), p53, and NF-κB.

Quercetin and its derivatives act on a multitude of targets involved in the initiation and promotion/progression phases of CRC carcinogenesis, as described in established cell lines and other animal models of the disease. Among the anti-carcinogenic activities of quercetin, the most notable described in CRC are inhibition of cellular proliferation and growth, cell cycle arrest, induction of apoptosis, reduction in tumor size, decrease in number of tumor nodule, suppression of metastasis, decrease in inflammation, decrease in ROS (i.e., antioxidant activity), and reduction in multidrug resistance. Well documented in cell culture and rodent studies, the mechanisms of action and targets of quercetin mainly involve members of the pathways Wnt/β-catenin, PI3K/AKT/mTOR, MAPK/Erk, MAPK/JNK, MAPK/p38, p-53, and NF-κB (Figure 4), [58,59,60,61].

Nevertheless, the beneficial effects of quercetin are not yet strongly supported by clinical trials carried out in patient populations with documented CRC pathology. There is only one clinical trial to be found by our team in the scientific literature advocating for the role of quercetin as chemopreventive agent. This study was carried out by Cruz-Correa and colleagues in 2006, involving five patients with prior colectomy and familial adenomatous polyposis [74]. After 6 months of supplementation regimen with 60 mg quercetin and 1.44 g curcumin per day, a reduction in the number and size of adenomatous polyps was observed in the study population [74]. The aforementioned preliminary data appear encouraging, aligning clinical and laboratory research on the activity of quercetin in pathologies such as CRC. In what concerns other studies in the field, there are only a few inconclusive results available regarding the effect of quercetin intake on colorectal cancer development described in statistical population studies [75,76,77]. However, the available clinical results can be used as a predictor entailing that the translation of quercetin targets from model organisms to patients with CRC pathology will open promising new therapeutic avenues in the field.

## 5. Crucial Signal Transduction Pathways in CRC

There is an interplay between intra- and intercellular factors that regulates physiological and spurs pathological processes, with signal transduction pathways at its core. In CRC development, although the signaling cascades are implicated in the modulation of distinct oncogenic mechanisms, they are finely tuned mainly by feedback mechanisms and both upstream and downstream common inducers and effectors [78].

### 5.1. Wnt/β-Catenin Signaling in CRC

The critical role of the Wnt/β-catenin pathway (Figure 4) in the etiology of CRC has been thoroughly studied; thus, light has been shed on the molecular mechanisms of interaction and the signal transduction regulation. Genetic alterations in the members of Wnt/β-catenin pathway lead to the intrinsic aberrant canonical Wnt/β-catenin activation, mainly stemming from mutations in the *APC*, *AXIN1*, and *AXIN2* genes [78].

Physiologically, β-catenin levels are maintained at sub-critical levels through the dynamic activity of the degradosome complex, consisting of the glycogen synthase kinase 3 (GSK3), axis inhibition protein 1 (AXIN1), adenomatous polyposis coli (APC), E3-ubiquitin ligase β-TrCP, protein phosphatase 2A (PP2A), and casein kinase 1α (CK1α) [79]. While the scaffold proteins in the complex are APC and AXIN1, the CK1α and GSK3 are responsible for β-catenin phosphorylation as serine/threonine kinases [80]. Once phosphorylated, E3-ubiquitin ligase β-TrCP mediates its ubiquitination and targets it for degradation by the proteasome machinery [78,81].

Pathologically, the Wnt ligands bind the 7-transmembrane receptor frizzled (FZD) family and its co-receptors low-density lipoprotein receptors 5 and 6 (LRP5/6) [82,83]. The ligand-receptor complex Wnt-FZD-LRP5/6 assembly with recruitment of the Dishevelled (DVL) adaptor by FZD facilitates the phosphorylation of LRP6 [84]. This leads to a cascade of molecular interactions including its association with AXIN1, their translocation to the plasma membrane, the dissociation of GSK3 from AXIN1 and APC, and concomitant stabilization of β-catenin by dephosphorylation [84,85]. Thereafter, the signalosome is assembled, a multiprotein complex that transduces Wnt signals, and the degradosome is disassembled leading to β-catenin accumulation in the cytosol and its subsequent nuclear translocation [84]. Nuclear β-catenin acts as a transcriptional activator inducing the transcription of target genes, among which are c-MYK [86] and AXIN2 [87], hereafter activating the oncogenic mechanisms [78].

### 5.2. PI3K/AKT-mTOR Signaling in CRC

Another relevant signal transduction pathway in CRC development and progression is the PI3K/AKT/mTOR cascade (Figure 4). It tightly interacts with the previously mentioned Wnt/β-catenin pathway, as blockage of, more accurately, PI3K/AKT/mTORC1 leads to hyperactivation of the Wnt/β-catenin as compensatory mechanism (Figure 3) [78].

The serine/threonine protein kinase mTOR consists of two multiprotein complexes: mTORC1 and mTORC2 [88], with the regulatory-associated protein of mTOR and the proline-rich AKT substrate of 40 KDa (PRAS40) being distinctive for the mTORC1 complex [89,90] and the rapamycin-insensitive companion of mTOR, the protein observed with RICTOR 1/2, and the mammalian stress-activated protein kinase-interacting protein 1 being distinctive for the mTORC2 complex [91,92,93]. While the molecular functions of mTORC2 were not fully elucidated, mTORC1 has been attributed several functions, some of which are vital pivots in the development of CRC. The most relevant upstream regulators of *mTORC1* suffering genetic alterations in the context of CRC are: *PIK3CA* gene-gain-of-function mutations [94,95], *PTEN* gene-inactivating mutations [95], or the *STK11/LKB1* gene [96], which encodes for an mTORC1 repressor. CRC reported mutations in the *mTOR* genes themselves are not as ubiquitous, while in the same pathway, the most seldom are the *AKT* gene mutations [97].

The mitogenic stimuli PI3K and AKT are the main activators of mTORC1 in the pathological context of CRC [98]. PI3K enzyme catalyzes the conversion of phosphatidylinositol (3,4)-bisphosphate into phosphatidylinositol (3,4,5)-trisphosphate, therefore triggering the phosphorylation of AKT [99]. Then, the AKT-mediated phosphorylation of mTORC1 takes place. This entails the phosphorylation of two main downstream targets: the eukaryotic translation initiation factor 4E (eIF4E) binding protein 1 (4E-BP1) and the S6 kinase 1 [100,101]. It is followed by 4E-BP1 dissociation from eIF4E, leading to mRNA translation activation, whilst S6K1 activation facilitates the phosphorylation of the S6 ribosomal protein leading to initiation and elongation of translation [102].

### 5.3. MAPK Cascades in CRC

The mitogen-activated protein kinase (MAPK) cascades (Figure 4) represent a set of membrane-to-nucleus signaling pathways that result in phosphorylation and activation of transcription factors [103]. With MAPK being members of the Ser/Thr kinases family, multiple rounds of subsequent phosphorylation-activating kinases are triggered [104].

There are three distinct MAPK cascades: MAPK/Erk (extracellular-signal-regulated kinases), MAPK/JNK (c-Jun N-terminal or stress-activated protein kinases), and MAPK/p38 [104]. As all pathways can be targeted (up- and down-regulated) by quercetin in the context of CRC, they will be the further discussed in the current publication.

Several proto-oncogenes are responsible for the involvement of the MAPK cascades in the development of CRC. The aberrations include the gain-of-function *KRAS* and *BRAF* gene mutations and upregulation of *JUN* gene [105]. Moreover, the role of EGFR is noteworthy in the MAPK activation and upregulation relevant in CRC [105].

#### 5.3.1. MAPK/ERK Signaling in CRC

EGFR, a transmembrane protein, member of the ErbB family of receptors, functions as a receptor tyrosine kinase located upstream of MAPK pathways [104]. The three Ras small GTPases are H-Ras, N-Ras, and K-Ras [106], while the most relevant Raf kinases are A-Raf, B-Raf and C-Raf (Raf1) [107]. Through the phosphorylation of the inactive form of Ras-family GTPases bound to GDP to the active form bound to GTP, external signals are transmitted from receptors on the cytoplasmic membrane to the interior of the cell [108]. The adaptor complex then activates Ras-GTP. Post RAS activation, there is a phosphorylation-dependent cascade, activating RAF, MEK, and, finally, ERK. The activation of the ERK/MAPK pathway is reported to also induce the synthesis of cyclin D1, relevant in the progression of cell cycle [109]. Moreover, Raf1 creates a link between the MAPK/ERK pathway and PI3K/AKT, allowing the possibility of correlated feedback between the two distinct metabolic paths, both upregulated in the CRC pathology [105].

#### 5.3.2. MAPK/JNK Signaling in CRC

The JNK cascade is used by the transforming growth factor-β (TGFβ) in order to autoregulate its concentration; however, it does not affect the JNK protein expression [110]. The pathway activation induced by TGFβ can act in conjunction with SMADs or in SMAD-independent manner [103,105]. The latter is acting on the MKK4–TGFβ-activated kinase 1 (TAK1) axis [110,111], where TAK1 is activated by the tumor necrosis factor-receptor-associated factor 6 (TRAF6), TGFβR2, and TGFβR1 protein complex [110]. Thereafter, it activates the JNK/p38 pathways [110], culminating in the formation of the ICD, domain of TGFβR1, consequent to ubiquitination and TNF-alpha converting enzyme cleavage [104]. ICD is able to translocate into the nucleus, where it induces the overexpression of *Snail*, *MMP2,* and *p300* genes [112].

#### 5.3.3. MAPK/ p38 Signaling in CRC

The MAPK/p38 pathway becomes activated in response to stressors such as hypoxia, heat shock, and osmotic shock [104]. p38 signaling is required for cell migration and metastasis in both CRC and breast cancer [113,114]. Similar to JNKs, p38 MAPKs are activated through autophosphorylation by MKKs [110,111]. TAK1 and TRAF6 are responsible for the SMAD-independent activation of p38. At this level, there is crosstalk in between TAK1 and the NF-κB-MMP9 pathway, another relevant carcinogenesis pathway reported in CRC. The blockade of p38 MAPK activity leads to the recovery of cell cycle and induction cell death mainly through autophagy [114,115].

### 5.4. p53 Signaling in CRC

*TP53*, a tumor suppressor gene, is among the most commonly mutated genes in CRC and various other types of cancer, with mutations mainly in the exons 5 to 8 (DNA binding domain) [116,117,118]. p53 is physiologically expressed at low levels, partly due to the negative feedback loops that involve MDM2. MDM2 is a transcriptional target of p53 that mediates the degradation of p53 through negative feedback and by functioning as an E3 ubiquitin-ligase that regulates the ubiquitination of p53 [119,120]. Low levels of p53 expression maintain homoeostasis of the cell cycle and cell death. A homolog of MDM2, namely, MDM4, not regulated by p53, forms heterodimers with MDM2 and can enhance MDM2 induced p53 degradation [119]. As a response to stress factors, such as oncogenes, DNA damage, UV irradiation, free radicals, hypoxia, or deficiencies in nutrients and growth factors, p53 is also activated (Figure 4). Then, it can either repress or transactivate downstream targets that regulate cell cycle arrest, apoptosis, DNA repair, and angiogenesis and metastasis [121]. Upon activation, under normal conditions, it can trigger both the intrinsic, mitochondrial, and the extrinsic, death-receptor-induced, apoptotic pathways [122]. The upregulation of expression takes place for the pro-apoptotic B-cell lymphoma-2 (Bcl-2) proteins, such as Bax, Noxa, and PUMA, while the pro-survival Bcl-2 members are downregulated, under normal conditions. This leads to the permeabilization of the mitochondrial outer membrane, releasing cytochrome-c, which binds to Apaf-1, activating the caspase-9. Thereafter, caspase-9 acts as initiator of the cascade, activating caspase-3, caspase-6, and caspase-7 [123]. Among the p53 upregulated death receptors, the most relevant would be PIDD (p53-induced protein with death domain), DR5 (TRAIL-R2), and Fas (CD95/APO-1), which alongside caspase-8 form the death-inducing signaling complexes acting in a loop and in turn activate p53 [120]. Moreover, the transcription factor (TF), *TP53*, is additionally involved in the genetic modulation including several miRNAs [116,121]. In the cell cycle, under normal conditions, p53 induces the G1/S and G2/M arrest via interactions with targets such as p21(WAF1), GADD45, retinoblastoma protein (Rb), and 14-3-3σ, also cRRIMA-1^MET^ [120].

### 5.5. NF-κB Signaling in CRC

NF-κB is a heterodimer protein, consisting of the p65 and p50 subunits, which are required for its activation and translocation to the nucleus (Figure 4) [124,125]. Physiologically, in most quiescent cells it is retained in the cytoplasm by I-kappa B (IκB), which covers its nuclear localization sequence (NLS) [126]. Pathologically, the IκB kinase (IKK) complex, containing the NEMO regulatory subunit and the IKKα and IKKβ catalytic subunits, is upregulated by external stimuli through receptors such as the tumor necrosis factor receptor (TNFR), the Toll-like receptor (TLR), and the T/B cell receptor [124,127]. In turn, it phosphorylates IκB, which then is degraded via the ubiquitin-proteasome pathway, allowing thereafter the nuclear translocation of NF-κB [124]. Inside the nucleus, it triggers down-stream gene expression by binding to the enhancer element of the immunoglobulin kappa light-chain, leading to inflammation and cancer development or progression [127,128,129]. In CRC adenocarcinoma, the abnormal activity of K-RAS is directly proportional with the expression of NF-κB [130].

## 6. Quercetin Impacts the Growth and Proliferation in CRC

In the development of CRC, cellular growth and proliferation mechanisms need to be altered for the progression of the disease. It is noteworthy that quercetin is documented, in Table 3, as an inhibitor of these processes both in vivo and in vitro [131,132,133,134,135,136,137,138,139,140,141]; however, the molecular mechanisms are not fully understood due to its multitude of targets and, quite often, lack of analysis beyond the mere description of the phenomenon [136,137,138,139,140,141].

Quercetin inhibits in cell culture the activity of AKT (also known as protein kinase B) by hindering it from phosphorylation, thus decreasing the concentration of p-AKT, in several CRC representative cell lines, such as HT-29 [131,133], Caco-2 [132], DLD-1 [132], and HCT-15 [133]. This is a counteractive mechanism to the hyperactivation of the PI3K/AKT signal transduction pathway in the context of CRC development, presented in the previous chapter. Similarly, the inhibitory multi-target action of quercetin on the other members of the PI3K/AKT is noted at the level of the PI3K, S6, and 4E-BP1 in studies conducted Caco-2 and DLD-1 cell lines [132]. In line with the expected anti-carcinogenic properties of the substance, quercetin is also modulating the Wnt/β-catenin signaling pathway, qualitatively demonstrated through the decrease in p-GSK3β concentration in Caco-2, DLD-1, HT-29, and HCT-15 cell lines [132,133], fighting against the constant activation of the pathway as described in diseased CRC models. A decrease in MYC and cyclin D1 is expected, however, also quantified, upon downregulation of the Wnt/β-catenin pathway in the HT-29 cell line upon media supplementation with quercetin [131,133]. STAT3 is a molecule that requires for its phosphorylation a third upregulated pathway in CRC, namely, the MAPK cascades [140]. It is also among the indirect targets of quercetin, the concentration of p-STAT3 decreasing upon media supplementation with the flavonol for the Caco-2 and DLD-1 cell cultures [132].

In vivo, Wistar rats and F344 rats, studies claim a decrease in proliferating cell nuclear antigen (PCNA) and annexin A1 (ANXA1) upon dietary ingestion of quercetin in comparison to the control group [134,135]. PCNA, originally a DNA sliding clamp for replicative polymerases and vital component of the eukaryotic chromosomal DNA replisome, has been revealed to interact with multiple partners, involved in DNA repair, Okazaki fragment processing, DNA methylation, and chromatin remodeling [142]. ANXA1, also known as lipocortin I, is a member of the annexin multigene superfamily of Ca^2+^-regulated, phospholipid-dependent, membrane-binding proteins [143]. In CRC, ANXA1 upregulation is correlated with the MAPK cascades upregulation, as its concentration is directly proportional with the K-RAS concentration [143,144,145].

## 7. Quercetin Impacts the Cell Cycle in CRC

Even though highly related to the previously described section, the targets of quercetin in the cell cycle arrest might slightly differ, while the activity is mainly described regarding the phase in which the cells are resting (Table 4).

While only one study on the effect of quercetin supplementation in the context of CRC was found in scientific literature [146], more attention was dedicated to the cell culture models [131,138,147,148,149,150,151,152]. In vivo, G0/G1 arrest was observed for HCT-116 cells grafted as xenograft in a mouse model [146]. However, no further biochemical analysis was conducted to reveal the molecular factors responsible. Nevertheless, in the same G0/G1 phase HT-29 cells were arrested upon quercetin media supplementation in vitro [131], while HCT-116 as non-grafted cell line was arrested in G1 or G2 [138]. Most studies, however, have noted a G2/M arrest in HT-29, HCT116, SW480 [147], RKO [148], and SW620 [149].

**Table 4:** Reported targets of quercetin active in cell cycle arrest of CRC models alongside their in vivo/in vitro testing system. “↑” and “↓” arrows are indicating the up- and downregulation, respectively, while “?” denotes the lack of specific targets in the respective studies.

Cell Cycle Arrest Phase and/or Molecular Targets	Testing System	Reference
At G0/G1 phase	In vivo: HCT-116 Xenograft mouse model	[146]
?		
At G0/G1 phase	In vitro: HT-29 cell culture	[131]
?		
At G1 or G2	In vitro: HCT-116 cell culture	[138]
?		
At G2/M	In vitro: HT-29, HCT116 and SW480 cell cultures	[147]
↓ p-AKT		
↑ Cyclin B1		
At G2/M	In vitro: RKO cell culture	[148]
↓ CDK1, CDC25c, Cyclin B1		
↑ p21		
At G2/M	In vitro: SW620 cell culture	[149]
↑ p21, p58		
↓ CDC6, CDK4, Cyclin D1	In vitro: Caco-2 cell culture	[151]
↓ Ki67	In vitro: SW480 cell culture	[152]
↓ Bcl-2	In vitro: HT-29 cell culture	[131]
↑ Bax, p53, Caspase-3		

Abbreviations: Bax = Bcl-2 Associated X-protein; Bcl-2 = B-cell lymphoma 2; CDC6 = Cell division cycle 6 regulatory protein; CDC25c = Cell division cycle 25c regulatory protein; CDK1 = Cyclin dependent kinase 1; CDK4 = Cyclin dependent kinase 1; Ki67 = nonhistone nuclear protein KI67; p-AKT = phosphorylated Protein kinase B; p21 = Cyclin-dependent kinase inhibitor 1; p53 = Tumor protein p53; p58 = p58 Natural killer cell inhibitory receptor.

While down regulation of AKT was already discussed in this context [147], the effect of quercetin on the cyclin B1 has been observed to be contradictory upon media supplementation with quercetin [147,148]. This could be explained by the physiological tight regulation and significant changes in the cyclin B1 during the cell cycle, especially in the G2/M phase [150]. Moving on to another cyclin, inhibition of cyclin D1, shows the same impact of quercetin as previously described [151]. In what concerns the p53 pathway members, p53, Bax, caspase-3, and Bcl-2 are counter-regulated by quercetin as compared to their expression in the context of the CRC pathology [131].

## 8. Quercetin Impacts Apoptosis in CRC

Alongside suppression of proliferation, induction of apoptosis increases the theoretical benefits of an anti-cancer agent. In this regard, quercetin takes both approaches against cancer development, with a plentitude of targets, mainly pertaining to signal transduction pathways (Table 5). Among the in vitro and the in vivo studies reviewed, quercetin mainly approaches through downregulation the pro-survival Bcl-2 component of the p53 pathway [134,136,148,153,154], the members of the PI3K/AKT/mTOR, Wnt/β-catenin, NF-κB and MAPK signaling pathways [135,136,152,153,155], the MMPs [153,156,157], the anabolism with AMPK as a marker [146,158,159], and the stress response to ROS [149]. It, however, concomitantly, upregulates the members of the p53 apoptotic cascade [133,134,136,137,140,148,149,152,153,154,155,160,161,162], and cell-to-cell adherence inhibition E-cadherin [134,153]. Unexpected upregulations are observed for two MAPK cascades, namely, JNK and p38, in HCT-15 [154], HCT-116 [157], Caco-2, DLD-1 [132], DLD-1^KRASG13D^, and DLD-1^KRASWT^ [137] cell lines under experimental conditions. 

## 9. Quercetin Impacts Tumor Size in CRC

In contrast to the previous section describing quercetin-induced apoptosis, the impact of the phytochemical on tumor size was solely described in vivo, due to clear limitations of cell culture system in this pursuit (Table 6). Here, reported CRC models were rodents, namely, mice and rats [139,152,153,162]. In the case of the PI3K/AKT and p53 pathways, the quercetin-induced modulation is in accordance with its expected anticarcinogenic activity [152]. However, in the case of the MAPK cascades, the modulation seems to act in anti-apoptotic and pro-proliferative manner, even though the quantification of the tumor size contradicts this in the testing system [153]. Nevertheless, all studies attest that the tumors decreased in size upon quercetin supplementation, supporting, through an additional argument, the potential benefits of testing quercetin alongside chemo- and radiotherapy in clinical setting. 

## 10. Quercetin Impacts Tumor Nodule Number in CRC

From the perspective of the tumor nodule number, quercetin has been observed to positively impact the prognosis of the tested rodents [139,162,163,164]. However, the molecular mechanism was not monitored in the reviewed studies (Table 7). In line with previously described pro-apoptotic and anti-proliferative actions of quercetin, this beneficial activity of the substance is plausible and could bring benefits to be further explored for future management of CRC patients.

## 11. Quercetin Impacts Migration and Invasion in CRC

While the impact of quercetin in tumor size reduction and decrease in tumor nodule number were reported exclusively in vivo, it could be surprising that its function in the suppression of migration and invasion is only reported in vitro [165,166]. However, the compelling aspect of the reviewed data lays in the monitorization of molecular targets influenced by the presence of quercetin (Table 8).

Both studies report quercetin induction of E-cadherin, which has a vital role in cellular contact inhibition [165,166]. E-cadherin, component of the adherent junctions, binding between cells is important in mediating the contact inhibition of proliferating cells as they reach confluence [167]. Downregulation of E-cadherin results in the decrease in contact inhibition and, consequently, the increase in cell motility and advancement through the stages of cancer [167].

Moreover, the matrix metalloproteinases (MMPs) are a family of zinc-dependent endopeptidases [168]. Elevated levels of distinct MMPs are detected in tumor tissue or serum of patients with advanced cancer, and they are the major prognostic indicators in cancer [168]. MMP2 and MMP9, also known as gelatinases A and B, play a key role in the proteolytic cascade that leads to ECM cleavage during metastasis in patients with CRC pathology [169].

## 12. Quercetin Impacts Inflammation in CRC

Pro-inflammatory conditions promote the development of CRC; therefore, it is highly relevant that quercetin has anti-inflammatory potential in CRC models (Table 9), [135,154,170,171]. It is, however, contradictory that one study in the SW620/Ad300 cells grown in quercetin-supplemented media reports increase in ROS [170], as quercetin acts as both direct and indirect radical scavenger [21,172]. SLC1A5 is a Na^+^ dependent antiporter of neutral amino acids, with overexpression in proliferating immune cells [173], there supporting the in vitro claim linking quercetin, its downregulation, and an anti-inflammatory response [170]. The pro-inflammatory cytokine, tumor necrosis factor-α (TNF-α) ligand of the TNF family, is predominantly produced by macrophages as well as tumor cells [174]. Its activity could be one of the main drivers of CRC progression [175]. Along with previous observations regarding its role in the escape from apoptosis, quercetin inhibition on TNF-α is, indeed, a major player in the fight against CRC [171]. Besides TNF-α, adjacent COX-2 and iNOS protein expression is also influenced by the NF-κB pathway, which is negatively modulated by quercetin in the fight against CRC [176]. This is in line with the in vivo studies confirming reduced COX-2 and iNOS presence upon diet supplementation with the phytochemical in Wistar rats [134].

## 13. Quercetin Impacts Oxidative Stress in CRC

In what concerns the highly quoted antioxidant activity of quercetin, it is noted that it works directly as a ROS scavenger [21,172]. Indirectly, quercetin has two distinctive paths of action. On the one hand, it can induce the production of glutathione used as hydrogen donor by the superoxide dismutase (SOD), which captures O^2−^, transforms it into H_2_O_2_, and further decomposes it to the non-novice H_2_O [177,178]. On the other hand, it can modulate the non-enzyme-dependent antioxidant defense system pathways leading to the decrease in levels of ROS [21]. This entails to the enhancement of PI3K/AKT, Nrf2, MAPK/NF-κB, and AMPK, while being responsible for the inhibition of JNK [21,179,180,181,182,183].

In the context of colorectal cancer, a study conducted in RKO and CCD841 cell cultures treated with hermin shows that quercetin and its derivative, 3,4-dihydroxyphenylacetic acid, are able to restore the cellular damage produced by ROS and prevent CRC initiation [140]. Hermin is a metabolite of myoglobin, produced after dietary meat intake, thus making the utilized study model highly relevant for the role of dietary quercetin in the prevention of CRC. In this study, the monitored activities of caspase 3, cytochrome-c, complex I, and complex II of the electron transport chain were restored upon diet supplementation with the two phytochemicals and contrasted to the results obtained in the presence of sulforaphane, a known CRC protective agent [140].

## 14. Quercetin Impacts Chemoresistance in CRC

While single-agent approaches are less likely to be effective, there is emerging evidence for the synergistic effect of polyphenols, showing a potentiating effect of chemotherapy in different types of cancer [184,185,186,187]. While a decrease in multi-drug resistance is quoted among the activities attributed to quercetin in CRC, there is little concrete evidence in the scientific literature. One study conducted in colon cancer SW620/Ad300 cells reveals that quercetin improves the cytotoxicity of doxorubicin [170]. This is achieved through inhibition of the ATP-driven transport activity of P- glycoprotein, leading to increased intracellular concentration of doxorubicin [170]. Moreover, the UPLC-MS/MS metabolomic analysis reveal that quercetin could reverse the multidrug resistance by blocking the D-glutamine and the D-glutamate metabolism, via down-regulation of the expression of the glutamine transporter solute carrier family 1, member 5 (SLC1A5) in SW620/Ad300 cells [170].

## 15. Conclusions

The postgenomic era brought about an unprecedented wealth of information regarding the human body. Interestingly, whenever we face a critical health condition, we tend to become more health conscious and advance our understanding; however, all that emerges is a puzzling picture that denotes an overwhelming complexity. Despite being unprepared for this outcome, we must continue to seek novel approaches to comprehend the complexity of life. In the current review, we surveyed quercetin and CRC in the scientific literature to elucidate the modulation of cancer-affected cellular mechanisms, in order to be better prepared for the prevention, rather than just treatment of the pathology.

Quercetin, a plant polyphenol abundantly found in many vegetables and fruits [31,32], up- and downregulates relevant pathways in the context of CRC, such as Wnt/β-catenin, PI3K/AKT/mTOR, MAPK/Erk, MAPK/JNK, MAPK/p38, p-53, and NF-κB signalling cascades [58,59,60,61]. Due to its multitude of induced and/or inhibited molecular targets assessed both in vivo and in vitro, the functions of quercetin in pathological conditions, such as CRC, could be worth studying since certain anti-cancer effects are prominent.

Since the assessed studies were carried out with isolated aglycan quercetin, a question of the possible implication of quercetin-rich plants remains open. In this respect, the avenues of nutrigenetics could also offer novel insights, so that not only single molecule caused effects, but the synergism of a combination of phytochemicals should also be assessed. Such studies could further substantiate the preventive nutrition that might offer plentitude of benefits. Moreover, the assessment of quercetin impact on CRC could bring about special dietary plan in the management of CRC patients as complementary therapy.

In the context of CRC and public health, this review points out the benefits of quercetin consumption in a molecular manner, while indirectly advocating for a healthier diet. With an increase in the toll of this pathology worldwide, it is worth widening our research efforts towards the potential inclusion of a quercetin dietary prevention.

## Figures and Tables

**Table 1:** Dietary sources with quercetin concentrations higher than 10 mg/100 g fresh weight according to the United States Department of Agriculture Database for the Flavonoid Content of Selected Foods.

Plant	Quercetin Concentration
	(mg/100 g Fresh Weight)
Dill	79.0
Fennel leaves	46.8
Onion	45.0
Oregano	42.0
Chili pepper	32.6
Spinach	27.2
Cranberry	25.0
Kale	22.6
Cherry	17.4
Lettuce	14.7
Blueberry	14.6
Asparagus	14.0
Broccoli	13.7
Chives	10.4

Source: USDA (United States Department of Agriculture) Database for the Flavonoid Content of Selected Foods [32].

**Table 2:** Selected quercetin derivatives alongside the position of the modification (see Figure 1 for correspondent numbers of the carbon atoms).

Selected Quercetin Derivative	Chemical Structure	Modification on A-Ring	Modification on B-Ring	Modification on C-Ring
Quercetin	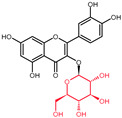	-	-	3-OH to 3-O- glucoside
3-O-glucoside				
(Isoquercetin)				
Quercetin	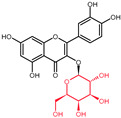	-	-	3-OH to 3-O- galactoside
3-O-galactoside				
(Hyperoside)				
Quercetin	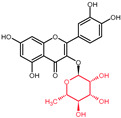	-	-	3-OH to 3-O- rhamnoside
3-O-rhamnoside				
(Quercitrin)				
Quercetin	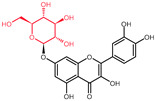	7-OH to 7-O-glucoside	-	-
7-O-glucoside				
(Quercimeritrin)				
Quercetin	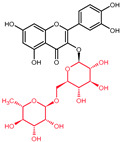	-	-	3-OH to 3-O- rutinoside
3-O-rutinoside				
(Rutin)				
Quercetin	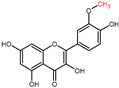	-	3′-OH to 3′-methyl ether	-
3-methyl ether				
(Isorhamnetin)				
Isorhamnetin	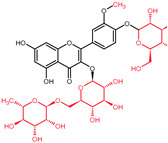	-	3′-OH to 3′-methyl ether 4′-OH to 4′-O-glucoside	3-OH to 3-O- rutinoside
3-O-rutinoside-				
4′-O-glucoside				
Isorhamnetin	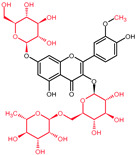	7-OH to 7-O-glucoside	3′-OH to 3′-methyl ether	3-OH to 3-O- rutinoside
3-O-rutinoside-				
7-O-glucoside				
Quercetin	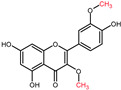	-	3′-OH to 3′-methyl ether	3-OH to 3-methyl ether
3,3′-dimethyl ether				
Quercetin	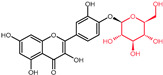	-	4′-OH to 4′-O-glucoside	-
4′-O-glucoside				
(Spiraeoside)				
Quercetin	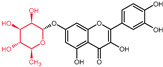	7-OH to 7-O-rhamnoside	-	-
7-O-rhamnoside				
Quercetin	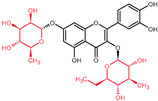	7-OH to 7-O-rhamnoside	-	3-OH to 3-O- glucoside
3-O-glucoside-				
7-O-rhamnoside				
(VincetoxicosideA)				
4′-O-methyl	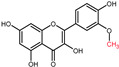	-	4′-OH to 4′- methyl ether	-
quercetin				
(Tamarixetin)				
7-O-methyl	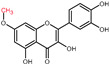	7-OH to 7-methyl ether	-	-
quercetin				
(Rhamnetin)				
3′, 7-dimethyl quercetin	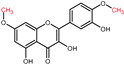	7-OH to 7-methyl ether	3′-OH to 3′-methyl ether	-
(Rhamnazin)				

Chemical structure source: *PubChem* [41].

**Table 3:** Reported targets of quercetin active in the reduction in cellular growth and proliferation of CRC models alongside their in vivo/in vitro testing system. “↓” arrows are indicating the downregulation, while “?” denotes the lack of specific targets in the respective studies.

Molecular Targets	Testing System	Reference
↓ p-AKT, MYC	In vitro: HT-29 cell culture	[131]
↓ CB1 receptor, Wnt/β-catenin, p-GSK3β,	In vitro: Caco-2 and DLD-1 cell cultures	[132]
p-PI3K, p-AKT, p-S6, p-4E-BP1, p-STAT3		
↓ p-AKT, p-GSK3β, Cyclin D1	In vitro: HT-29 and HCT-15 cell cultures	[133]
↓ PCNA	In vivo: Wistar rats	[134]
↓ ANXA1	In vivo: F344 rats	[135]
?	In vitro: HCT-116 and HT-29 cell cultures	[136]
?	In vitro: DLD-1^KRASG13D^, DLD-1^KRASWT^, SW480^KRASG12V^, HCT-116^KRASG13D^, Colo205^KRASWT^, WIDR^KRASWT^, and HT-29 ^KRASWT^ cell cultures	[137]
?	In vitro: HCT-116 cell culture	[138]
?	In vitro: HCT15 and CO115 cell cultures	[135]
?	In vitro: HCT-116 cell culture	[139]
?	In vitro: RKO and CCD841 cell cultures	[140]
?	In vivo: F344 AOM treated rats	[141]

Abbreviations: ANXA1 = Annexin A1; CB1 receptor = Cannabinoid receptor type 1; MYC = Myelocytomatosis oncogene product; p-4E-BP1 = phosphorylated Eukaryotic translation initiation factor 4E binding protein 1; p-AKT = phosphorylated Protein kinase B; p-GSK3β = phosphorylated Glycogen synthase kinase 3 beta; p-PI3K = phosphorylated Phosphoinositide 3-kinase; p-S6 = phosphorylated Ribosomal protein S6; p-STAT3 = phosphorylated Signal transducer and activator of transcription 3; PCNA = Proliferating cell nuclear antigen; Wnt/β-catenin = Wingless-related integration site/β-catenin pathway.

**Table 5:** Reported targets of quercetin active in induction of apoptosis of CRC models alongside their in vivo/in vitro testing system. “↑” and “↓” arrows are indicating the up- and down-regulation, respectively, while “?” denotes the lack of specific targets in the respective studies.

Molecular Targets	Testing System	Reference
↑ JNK, c-Jun	In vitro: Caco-2 and DLD-1 cell cultures	[132]
↑ COX-2	In vitro: HT-29 and HCT-15 cell cultures	[133]
↑ Caspase-3, Cytochrome-c		
↑ Bax, PARP, APC	In vivo: Wistar rats	[134]
↓ Bcl-2, β-catenin		
↓ p-ERK, KRAS	In vitro: HCT-15 cell culture	[135]
↓ TSC22 domain family 3	In vivo: F344 rats	[135]
↓ p-AKT, KRAS	In vitro: CO115 cell culture	[135]
↓ PI3K, AKT, p-AKT, Bcl-2	In vitro: HCT-116 and HT29 cell cultures	[136]
↑ Bax		
↑ Caspase-3, p-JNK	In vitro: DLD-1^KRASG13D^ and DLD-1^KRASWT^ cell cultures	[137]
↓ p-AKT		
↑ Caspase-3, Cytochrome-c	In vitro: RKO and CCD841 cell cultures	[140]
↓ AMPK, HIF-1	In vitro: HCT-116	[146]
↓ Bcl-2	In vitro: RKO cell culture	[148]
↑ Bax, cleaved-Caspase-3, cleaved-Caspase-9		
↑ Bax, Cytochrome-c, Caspase-9, Apaf-1, Caspase-3	In vitro: SW620 cell culture	[149]
↓ GPx, Catalase		
↓ PI3K, AKT ↑ Caspase-3, Bax	In vitro: SW480 cell culture	[152]
↑ PARP, cleaved-Caspase-3, cleaved-Caspase-9	In vitro: CT-26 cell culture	[153]
↓ Bcl-2, Bcl-xL		
↓ MMP-2, MMP-9, N-cadherin, β-catenin, Snail	In vivo: mouse model of CRC lung metastasis	[153]
↑ E-cadherin		
↑ p53, BAX, p-p38 ↓ Bcl-2	In vitro: HCT-15 cell culture	[154]
↑ p53, cleaved-Caspase 3, cleaved-Caspase 9, PARP, cleaved-PARP ↓ Bcl-2	In vitro: CO115 cell culture	[154]
↑ Bax, Caspase-3, Caspase-9	In vitro: Caco-2 and SW-620 cell cultures	[155]
↓ Bcl-2, NF-κB		
↓ MMP	In vitro: DLD-1 cell culture	[156]
↓ MMP	In vitro: HCT-116 cell culture	[157]
↑ SIRT-2, p-AMPK, p-p38	In vitro: HCT-116 cell culture	[158]
↓ p-mTOR		
↑ Caspase-3, cleaved-PARP, p-p38	In vitro: DLD-1 cell culture	[160]
↓ Bcl-2, Cyclin D1,	In vitro: Colo320 cell culture	[161]
↑ Bax, Caspase-3, Wnt1, Catalase		
?	In vivo: AOM/DSS-treated wild-type C57BL/6J mice	[162]
?	In vitro: HCT-116 cell culture	[138]
?	In vitro: HCT-116 cell culture	[141]
?	In vitro: HCT-116^p53-wt^, HCT-116^p53-null^, HCT-15^KRAS-mutated^ cell culture	[154]
?	In vitro: CT-26 cell culture	[163]

Abbreviations: AKT = Protein kinase B; AMPK = 5′ adenosine monophosphate-activated protein kinase; Apaf-1 = Apoptotic protease activating factor 1; APC = Adenomatous polyposis coli; Bax = Bcl-2 Associated X-protein; Bcl-2 = B-cell lymphoma 2; Bcl-xL = B-cell lymphoma extra-large; c-Jun = AP-1 transcription factor subunit; COX-2 = cyclooxygenase-2; GPx = Glutathione peroxidase; HIF-1 = Hypoxia-inducible factor 1; JNK = c-Jun N-terminal kinases; KRAS = Kirsten rat sarcoma virus; MMP = Matrix metalloproteinases; MMP-2 = Matrix metalloproteinase 2; MMP-9 = Matrix metalloproteinase 9; NF-κB = Nuclear factor kappa-light-chain-enhancer of activated B-cells; p-AKT = phosphorylated Protein kinase B; p-AMPK = phosphorylated 5′ adenosine monophosphate-activated protein kinase; p-ERK = phosphorylated Extracellular signal-regulated kinase; p-JNK = phosphorylated c-Jun N-terminal kinases; p-mTOR = phosphorylated Mammalian target of rapamycin; p-p38 = phosphorylated Mitogen-activated protein kinase p38; PARP = Poly (ADP-ribose) polymerase; PI3K = Phosphoinositide 3-kinases; SIRT-2 = NAD-dependent deacetylase sirtuin 2; Snail = Zinc finger protein SNAI1; TSC22 domain family 3 = Glucocorticoid-induced leucine zipper protein; Wnt1 = Proto-oncogene Wnt-1.

**Table 6:** Reported targets of quercetin active in the reduction in tumor size of CRC models alongside their in vivo testing system. “↑” and “↓” arrows are indicating the up- and down-regulation, respectively, while “?” denotes the lack of specific targets in the respective studies.

Molecular Targets	Testing System	Reference
↓ PI3K, AKT	In vivo: SPF grade BALB/C nude mice	[152]
↑ caspase-3, Bax		
↑ p-Erk, p-JNK, p-p38	In vivo: mouse model of CRC lung metastasis	[153]
?	In vivo: AOM/DSS-treated wild-type C57BL/6J mice	[162]
?	In vivo: F344 AOM-treated rats	[139]

Abbreviations: AKT = Protein kinase B; Bax = Bcl-2 Associated X-protein; p-ERK = phosphorylated Extracellular signal-regulated kinase; p-JNK = phosphorylated c-Jun N-terminal kinases; p-p38 = phosphorylated Mitogen-activated protein kinase p38; PI3K = Phosphoinositide 3-kinases.

**Table 7:** Reported targets of quercetin active in decreasing the number of tumor nodules of CRC models alongside their in vivo testing system. “?” denotes the lack of specific targets in the respective studies.

Molecular Targets	Testing System	Reference
?	In vivo: F344 AOM-treated rats	[139]
?	In vivo: AOM/DSS-treated wild-type C57BL/6J mice	[162]
?	In vivo: CT-26 mouse Xenograft model	[163]
?	In vivo: Apc^Min/+^ mice	[164]

**Table 8:** Reported targets of quercetin active in suppression of migration and invasion of CRC models alongside their in vitro testing system. “↑” and “↓” arrows are indicating the up- and downregulation, respectively.

Molecular Targets	Testing System	Reference
↑ E-cadherin	In vitro: Caco-2 cell culture	[165]
↓ MMP-2, MMP-9, TLR4, NF-ҡB, TNF-α, COX-2,IL-6		
↑ E-cadherin ↓ Twist1, Vimentin	In vitro: SW-480 cell culture [165]	[165]

Abbreviations: COX-2 = cyclooxygenase-2; IL-6 = Interleukin 6; MMP-2 = Matrix metalloproteinase 2; MMP-9 = Matrix metalloproteinase 9; NF-κB = Nuclear factor kappa-light-chain-enhancer of activated B-cells; TLR4 = Toll-like receptor 4, TNF-α = Tumor necrosis factor alpha.

**Table 9:** Reported targets of quercetin active in the reduction in inflammation of CRC models alongside their in vivo/in vitro testing system. “↓” arrows are indicating the downregulation, while “?” denotes the lack of specific targets in the respective studies.

Molecular Targets	Testing System	Reference
↓ COX-2, iNOS, NF-κB	In vivo: Wistar rats	[134]
↓ SLC1A5 glutamine transporter	In vitro: SW620/Ad300 cell culture	[170]
↓ TNF-α	In vivo: AOM/DSS-treated wild-type C57BL/6J mice	[171]
?	In vivo: AOM/DSS-treated wild-type C57BL/6J mice	[162]

Abbreviations: COX-2 = cyclooxygenase-2; iNOS = Inducible nitric oxide synthase; NF-κB = Nuclear factor kappa-light-chain-enhancer of activated B-cells; SLC1A5 = glutamine transporter solute carrier family 1, member 5; TNF-α = Tumor necrosis factor alpha.

## Data Availability

Not applicable.
